# Effect of claw blocks on the healing duration and lesion severities of claw lesions in lame cows in Western Thailand

**DOI:** 10.14202/vetworld.2023.258-263

**Published:** 2023-02-11

**Authors:** Pipat Arunvipas, Teerachad Setkit, Jaturong Wongsanit, Theera Rukkwamsuk, Nitipong Homwong, Anawat Sangmalee

**Affiliations:** 1 Department of Large Animal and Wildlife Clinical Sciences, Faculty of Veterinary Medicine, Kasetsart University, Kamphaeng Saen Campus, Nakhon Pathom, Thailand; 2 Department of Animal Science, Faculty of Agriculture at Kamphaeng Saen, Kasetsart University, Kamphaeng Saen Campus, Nakhon Pathom, Thailand

**Keywords:** claw block, claw lesion, dairy cows, lameness

## Abstract

**Background and Aim:**

Lameness is a major complication in dairy cattle affecting health and milk production. Several factors are found to contribute to this condition and specific treatments are required, including the process of claw trimming. The elevation of the claw, such as with the application of a claw block, was reported to be beneficial in the more severe cases. This study aimed to determine the efficiency of a claw block on claw lesions of lame cows in dairy farms in Western Thailand.

**Materials and Methods:**

Locomotion scores of 376 dairy cows were determined by a veterinarian using a scale of 1–5 (1 = normal; 5 = severely lame) at the time of the visit. Cows with a score of 3 or greater were defined as clinically lame. In total, 134 clinically lame cows from 11 dairy farms in the Kanchanaburi and Ratchaburi provinces were included in the analysis. Claw lesions included a white line abscess, bruised sole, sole ulcer, sole abscess, white line separate, and double soles. Wooden or rubber claw blocks were applied to the unaffected claw of the same hoof as the injured claw of 116 cows, which were classified as the treatment cases, and 18 cows were left untreated and classified as the control cases. Each cow was checked on every week of the healing process for 2 months unless the cow was culled earlier. Survival analysis was based on the Kaplan–Meier estimator and Cox Proportional Hazard regression.

**Results:**

The median healing time for lame cows with and without claw blocks was 21 and 36 days, respectively. After adjusting for the lesion severity and type, the lame cows with and without a claw block had hazard ratios of 2.16 and 3.08, respectively. The healing times between the four lesion types in cows with a claw block were not significantly different. The healing time was longer in lame cows, with a severity score of 4.

**Conclusion:**

The results from this study reveal that the treatment of lame cows with claw blocks promoted the healing capacity of claw lesions after claw trimming.

## Introduction

Lameness is a painful condition for cattle and is considered as one of the most important disorders in dairy cattle resulting in production and economic losses [1–3]. Lameness is recognized as the third most serious production disease in UK dairy farms and has adverse effects on both animal welfare [4] and health economics [5]. Most economic consequences caused by lameness result from the increase in involuntary culling [6], reduced milk yield [7, 8], and a long time from calving to conception and prolonged calving interval [9, 10]. Lameness in dairy cattle is a multifactorial condition and risk factors for specific foot and leg conditions have been identified in several studies. For example, the floor type, cubicle dimensions, parity, stage of lactation, and milk production have been associated with lameness in cattle [11, 12]. In some areas, the lack of a claw trimmer exacerbated lameness and increased the severity in lame cows [13–15]. Furthermore, claw disorders cause approximately 90% of lameness in dairy cattle [16]. A shorter time standing to eat makes cows weak, reducing milk production [17, 18], and resulting in a reduced life span for the affected dairy cows. Many factors are important in affecting claw quality.

A study involving Thai dairy farms found that the tie stall system was a risk factor for lameness, with the average prevalence in lactating cows being 22.0% and ranging from 0% to 70% [19]. A study involving Malaysian dairy farms reported an average lameness prevalence of 19.1%, ranging between 10.0%–33.3% [20]. Clarkson *et al*. [21] reported that training farmers to recognize early cases of lameness and to request veterinary treatment resulted in a marked reduction in the duration of lameness. Ratanapob *et al*. [10] showed that non-pregnancy in farms with a high prevalence of lameness and in lame cows could be reduced by 43%–70%, respectively, if lameness had been prevented.

A claw block is a treatment procedure for lameness, but information regarding its efficacy is limited. Therefore, this study aimed to determine the efficiency of claw blocks on claw lesions and lesion severity in lame cows on dairy farms in Western Thailand.

## Materials and Methods

### Ethical approval

This study was approved by the Animal Care and Use Committee for Scientific Research Committee, Kasetsart University, Bangkok, Thailand (ACKU61-VET-082).

### Study period and location

The study was conducted from July 2013 to February 2017. Dairy cows from the smallholder farms in Kanchanaburi and Ratchaburi provinces, Thailand, were included in this study.

### Animals

All the cows were in lactation number ranged from 1 to 3 and raised in either loose or tie stalls. The breed of the animals was Thai cross-bred Holstein-Friesian. The cows were fed on commercial concentrate feed and the roughage of choice available in the farm’s area such as corn silage and Napier grass. In total, 376 dairy cows were determined for their locomotion score at the time of routine visits by a veterinarian.

### Study design

Locomotion scores on a scale of 1–5 (1 = normal; 5 = severely lame) were assigned to each of the cows. Cows scoring 3 were defined as moderately lame, 4 as lame, and 5 as severely lame. Lameness was scored by adapting the 5-point scale into dichotomous categories of being lame or not lame, and cows scoring 3 or more were defined as clinically lame [22]. All lame cows underwent corrective and therapeutic trimming as needed. Lesions were recorded to include white line disease, white line separate, sole ulcer, sole abscess, bruised sole, double sole, median crack, and laminitis. The severity of each cow was classified using 5 levels: 0 = no lesions; 1 = lesions found and not deep to the corium; 2 = lesions found and deep to the corium; 3 = lesions found and deep to the corium with corium damage; and 4 = lesions found and passing through the corium to distal phalanx (P3).

All lesions were recorded and their areas were calculated using theImageJ program [23]. To determine “casecon” variables, a claw block was attached to the unaffected claw with a smooth sole and covering at least 50% of the sole area. Briefly, either wooden or rubber claw blocks were attached to the unaffected claw on the same hoof as the injured claw using glue or commercial adhesive. The adhesive was allowed to be completely set for approximately 10 min before the cow was released from the restraint. However, when there was less than 50% of the sole area available or the owner was unwilling to allow the use of a claw block, the cow was assigned to the control group (casecon = 0, without claw block), whereas casecon = 1 was the treatment group (with claw block). After trimming, the lesions of all cows were treated with iodine or tetracycline spray. In severe cases, the cows were treated with oxytetracycline (14 mg/kg) for 3 consecutive days. The lesion areas were then remeasured in cm^2^ using the ImageJ program [23] and locomotion scores were recorded based on the 5-point scale [22]. Depending on the measured time-to-healing based on locomotion scoring or lesion area, all cows were followed up weekly for at least 8 weeks unless they had returned to normal earlier or had been culled (defined as censored). A cow was defined as normal or healed if the sole growth had covered all lesions and no pain response was evident following percussion on the lesion and a locomotion score of 2 or less.

### Statistical analysis

Claw lesions were composed of white line disease, bruised sole, sole ulcer, sole abscess, white line separate, and double sole. Cows with claw block treatment were defined as cases, and cows without claw blocks were defined as controls. Cows were followed up every week during the healing process for 2 months unless the cow was culled, as this data was time-to-event. As previously stated, the severity was classified using a 5-point scale. Particular claw lesion scales with or without a claw block at visiting time were used to define the hazard rate (H), where H is a measure of the instantaneous risk of having a claw lesion (disease) at the given visiting time. Therefore, survival analysis based on the Kaplan–Meier Estimator and Cox Proportional Hazard regression was modeled and hazard rate ratios (HR) were estimated. According to the model, H was assumed to be constant over time but HR was not. In addition, during the preliminary analysis, the proportional hazard (PH) assumption was tested and it was found that the covariates did not change for the times that cattle became at risk of being lame; thus, the PH assumption was not violated. Hence, the general statistical model was used:

log(h(t|x*_i_*)) = log(h_o_(t)) + Σβ*_i_*x*_i_*

Where, β_i_ are regression coefficients; x_i_ are covariates I (i = 1, casecon, i = 2 severity, i = 3, lesion); and h_o_(t) is the baseline hazard (reference hazard) where x_i_ is the control group (without claw block) and no lesion.

The final model was selected based on the Akaike information criterion (AIC), where a lower AIC value indicates a better model. All statistical analysis was performed using the R version 4.0 software package [24] with the R “survival” package for Cox Proportional Hazard regression [25] and R “ggplot2” package [26] for graphical production. p < 0.05 was considered statistically significant.

## Results

From 376 observed dairy cows, 134 were determined to be clinically lame from 11 dairy farms and were included in the analysis. The results showed that, nine farms (76 cows) from Kanchanaburi Province and two farms (58 cows) from Ratchaburi province had lameness issues. The lesions were classified as white line disease (40.3%), sole abscess (32.09%), sole ulcer (14.8%), bruised sole (8.21%), white line separate (2.24%), median creak (1.49%), laminitis (0.75%), and double sole (0.75%). Claw lesions were more common in the median than lateral horns in both forelimbs (LF-Lat; 2.99%, LF-Med; 8.96%, RF-Lat; 2.99%, RF-Med; 7.46%). Lesions in the left and right hindlimbs were more common in the lateral than median horns (LH-Lat; 33.58%, LH-Med; 8.21%, RH-Lat; 31.34%, RH-Med; 3.73%). The proportions of the severity scale levels were: 1 (4.48%), 2 (17.16%), 3 (75.37%), and 4 (2.99%). Of the 134 clinically lame cows, 116 subsequently had claw blocks applied, while the remaining 18 cows were categorized into the control group, as previously mentioned.

Based on the AIC values for each model tested, Model 2 and Model 4 were the final models for predicting the measured time-to-healing using the locomotion score or lesion area, respectively (Table-1). Based on the locomotion score, the results of the final Cox Proportional Hazard model are summarized in Table-2. After adjusting for claw lesion severity, there was a significant association between time-to-healing and the use of claw block as a treatment intervention. Cows with a claw block had HR values as high as 3.081 for claw lesion healing compared to those withoutclaw blocks (HR values = 1). With a higher severity score, the use of claw block contributed significantly as the protective factor. Similarly, the results of the final Cox Proportional Hazard model for the measured lesion area are summarized in Table-3.

**Table-1:** Model selection for Cox Proportional Hazard models for locomotion score and lesion area.

Model	h (t|x_i_)	Casecon (x_1_)	Severity (x_2_)	Lesion (x_3_)	AIC	Final model
Locomotion score						
Model 1	Time-to-healing	√	×	×		
Model 2	Time-to-healing	√	√	×	682.16	√
Model 3	Time-to-healing	√	×	√	693.95	
Model 4	Time-to-healing	√	√	√	690.40	
Lesion area (healing status)						
Model 5	Time-to-healing	√	×	×	871.73	
Model 6	Time-to-healing	√	√	×	858.61	√
Model 7	Time-to-healing	√	×	√	878.33	
Model 8	Time-to-healing	√	√	√	868.97	

AIC: Akaike information criterion, measure of the goodness of fit and each model have a smaller value, indicating how well the proposed model fits the data.

**Table-2:** Results of final Cox Proportional Hazard model for the association between time-to-healing based on locomotion score and with- or without- claw block as a treatment intervention after adjusting for claw lesion severity.

Model 2	Hazard ratio	95% Confidence interval	p-value
Casecon			
Without a claw block as a control	1	-	-
With claw block as a case	3.081	1.499–6.377	0.002
Severity			
1	1	-	-
2	1.305	0.445–3.826	0.628
3	0.577	0.207–1.610	0.293
4	0.246	0.044–1.367	0.109

A significant level was considered at p < 0.05.

**Table-3:** Results of final Cox Proportional Hazard model for the association between time-to-healing based on lesion area and with- or without-claw block as a treatment intervention after adjusting for claw lesion severity.

Model 2	Hazard ratio	95% Confidence interval	p-value
Casecon			
Without a claw block as a control	1	-	-
With claw block as a case	3.028	1.634–5.612	<0.001
Severity			
1	1	-	-
2	1.066	0.430–2.641	0.890
3	0.581	0.249–1.356	0.209
4	0.047	0.005–0.433	0.007

A significant level was considered at p < 0.05.

**Figure-1:**
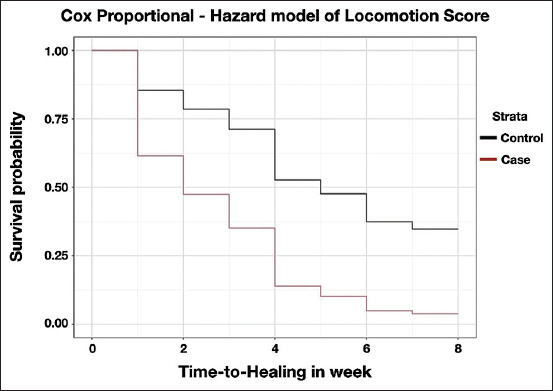
Survival curve estimated from final Cox Proportional Hazard model based on locomotion score after adjusting for severity.

**Figure-2:**
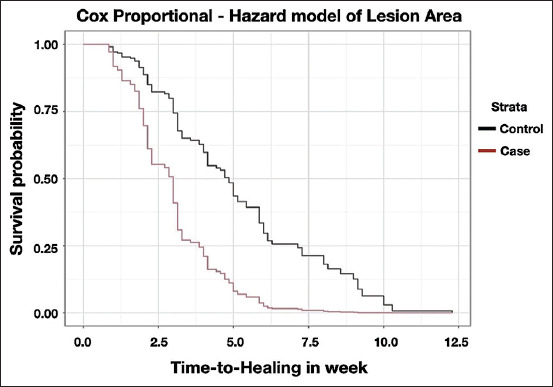
Survival curve estimated from final Cox Proportional Hazard model based on lesion area after adjusting severity.

The reduction in the survival function was proportional between the cases and controls, and the estimated survival curves based on the final Cox Proportional Hazard model (Model 2) were approximately parallel (Figure-1). The median survival time for the cases was 2 weeks after fitting the claw block, whereas the median survival time for the control was 5 weeks. Similarly, the reduction in the survival function was proportional between the cases and controls, and the survival curve estimated from the final Cox Proportional Hazard model (Model 6) was approximately parallel (Figure-2). The median survival time for cases was 2.8 weeks after fitting the claw block, whereas the median survival time for the controls was 4.7 weeks.

**Figure-3:**
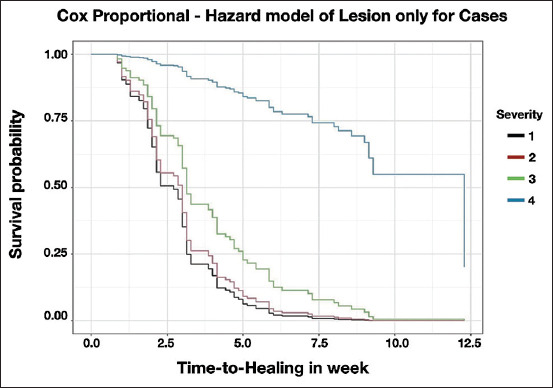
Survival curve of lesion severity estimated from final Cox Proportional Hazard model based on lesion area for only cases data with claw block compared to normal cows (severity = 0) with claw block as well.

Only the case group (with claw block) was analyzed from Model 6. The reduction in the survival function was proportional among severity levels 1, 2, and 3, and a survival curve was estimated (Figure-3). The median survival times after fitting the claw block for severity levels 1, 2, and 3 were 2.3, 2.9, and 3.0 weeks, respectively, whereas the median survival time for severity level 4 was 9.3 weeks.

## Discussion

The observations from this study are in agreement with the study conducted by Wongsanit *et al*. [19], where white line disease was reported as a significant lesion. Furthermore, a study in Selangor, Malaysia [20] reported sole ulcer (54.2%) and white line disease (61.9%) as two major lesions found in lame cattle. However, Murray *et al*. [16] reported slightly different results for claw lesions with the observed frequency of lesions being sole ulcer (28%), white line disease (22%), and digital dermatitis (13%). The previous reports have revealed different incidences of lameness [27, 28]. These differences may be due to various factors such as geographical and environmental variations of the study areas, the number of cases or samples, and differences in the definitions of claw lesion categories [29]. Several factors affecting hoof and claw lesions have been reported in many studies, including housing conditions, hard and slippery floors, and facility type, especially for animals raised in the tie stall system [19, 30], nutritional imbalance between roughage and concentrate feed [31], body conditions of the animals [28, 32], long-term exposure to a wet floor or moist conditions, a rough or sharp surface walkway, and the lack of a regular hoof trimming program [33, 34].

Claw lesions were more dominant on the median horns than lateral horns of both forelimbs. However, the lateral horns were a major factor regarding horns on the hindlimbs. Hindlimb lameness was more common than forelimb lameness in this study. These results are in agreement with many other reports [5, 16, 19, 35]. This was perhaps due to the anatomy of the cow claw and a stance where the weight distribution was higher on the forelimbs’ median horn than the hindlimbs’ lateral horn. Furthermore, the cattle hindlimbs were prone to exposure to moisture and dirt, such as feces and urine, as well as stepping on hard or sharp objects, as the hindlimbs can be considered a blind spot for cows while walking. The significant lameness severities in this study were scored as 3, where the lesions were deep to the corium and corium damage was recorded.

The healing duration of the claw lesions and severity between the treatment with claw block and control were investigated. Complete healing was defined as having no lesion area or a locomotion score of 2 or lower with no pain response when pressure was applied to the claws. The complete healing duration of the treatment group was approximately 21 days using both determination conditions. In comparison, the period was extended to 36 and 35 days by lesion area measurement and locomotion scoring, respectively, in the control group. The results confirmed the use of locomotion score as a routine farm-based lameness healing monitoring tool, which was previously reported as the method of choice for a lameness control program in the herd [36]. The average observed healing duration in this study was comparable with those from a study on sole ulcer healing time [37], where the average was 25 days without complications and approximately 45 days with evidence of complications. After adjusting for lesion severity, lame cows with no claw block had a higher HR of 3.08 (95% confidence interval, 1.49–6.38) than those with a claw block.

The healing duration in cows with claw blocks after claw trimming in this study was 3 times shorter (p < 0.0004) than the control group when the lesion severity was the same. Furthermore, the healing duration was 2.16 times shorter (p < 0.001) compared to the lesion types. This observation, together with the other studies [38, 39] confirms the significant advantage of combining the application of a claw block with the therapeutic trimming to heal claw lesions over conventional treatment trimming alone. This might be due to the benefit of the claw block on weight-bearing reduction and the relief of pressure and pain on the injured claw. Thomas *et al*. [40] also reported the benefit of combining NSAIDs administration with treatment trimming on the healing of lameness.

In this study, cows with a severity score of 4 had the most extended healing duration. The authors’ recommendation of culling was emphasized when the cow was neither pregnant nor produced high yields which is in agreement with a report by Van Amstel and Shearer [33]. Undiagnosed lameness in the herd leads to various issues, such as health, welfare, and production problems. The appropriate management and treatment at the causative level should serve as the key to successful lameness control. Other actions could include nutritional management, particularly of carbohydrate feed which could lead to rumen acidosis [41], avoiding rough walking surfaces, which could adversely affect hoof and claw health [31, 34, 41], and routine hoof and claw trimming to maintain claw conformation and weight bearing capability [19]. In addition, hoof and claw bathing with 2.5%–5% formalin solutions or 2.5% copper sulfate solutions were reported to be beneficial in addressing lameness problems [41]. Cook [31] reported evidence of a possible herd-level lameness problem if several cows showed clinical lameness. The early detection and treatment of lameness of cows in the herd are paramount to guaranteeing recovery from the disease and minimizing complications due to these problems. Claw elevation techniques have recently been considered a procedure to promote lameness healing, comprising a claw bandage or a wooden or rubber claw block [38, 39]. The rubber claw block was reported to be an uncomplicated and acceptable method recommended for farm-based practice.

## Conclusion

This study revealed that the four primary lameness lesions found in study animals of Western Thailand were white line disease, sole abscess, sole ulcer, and bruised sole. The lesions were categorized based on the degree of damaged claw tissue and the severity score was predominantly 3. Treatment of lame cows with a claw block aimed to promote the healing of claw lesions and to mitigate the hazard of lameness after claw trimming. In addition, locomotion scoring or measuring the lesion area directly was capable of evaluating the claw lesion healing.

## Authors’ Contributions

PA, TS, JW, TR, NH, and AS: Conceptualized and designed the study. TS: Conducted the experiments. PA: Performed the data analysis and drafted and edited the manuscript. JW, TR, and NH: Performed data analysis and interpretation. AS: Revised the manuscript. All authors have read and approved the final manuscript.
